# Anti-Cancer and Protective Effects of Royal Jelly for Therapy-Induced Toxicities in Malignancies

**DOI:** 10.3390/ijms19103270

**Published:** 2018-10-21

**Authors:** Yasuyoshi Miyata, Hideki Sakai

**Affiliations:** Department of Urology, Nagasaki University Graduate School of Biomedical Sciences, Nagasaki 852-8501, Japan; hsakai@nagasaki-u.ac.jp

**Keywords:** royal jelly, 10-hydroxy-2-decenoic acid, anti-cancer effects, toxicities, malignancies

## Abstract

Royal jelly (RJ) is a glandular secretion produced by worker honeybees and is a special food for the queen honeybee. It results in a significant prolongation of the lifespan of the queen honeybee compared with the worker honeybees through anti-inflammatory, anti-oxidant and anti-microbial activities. Consequently, RJ is used as cosmetic and dietary supplement throughout the world. In addition, in vitro studies and animal experiments have demonstrated that RJ inhibits cell proliferation and stimulates apoptosis in various types of malignant cells and affects the production of various chemokines, anti-oxidants and growth factors and the expression of cancer-related molecules in patients with malignancies, especially in patients treated with anti-cancer agents. Therefore, RJ is thought to exert anti-cancer effects on tumor growth and exhibit protective functions against drug-induced toxicities. RJ has also been demonstrated to be useful for suppression of adverse events, the maintenance of the quality of life during treatment and the improvement of prognosis in animal models and patients with malignancies. To understand the mechanisms of the beneficial effects of RJ, knowledge of the changes induced at the molecular level by RJ with respect to cell survival, inflammation, oxidative stress and other cancer-related factors is essential. In addition, the effects of combination therapies of RJ and other anti-cancer agents or natural compounds are important to determine the future direction of RJ-based treatment strategies. Therefore, in this review, we have covered the following five issues: (1) the anti-cancer effects of RJ and its main component, 10-hydroxy-2-decenoic acid; (2) the protective effects of RJ against anti-cancer agent-induced toxicities; (3) the molecular mechanisms of such beneficial effects of RJ; (4) the safety and toxicity of RJ; and (5) the future directions of RJ-based treatment strategies, with a discussion on the limitations of the study of the biological activities of RJ.

## 1. Introduction

The major therapeutic methods for patients with cancer are operations, chemotherapy and radiotherapy. In addition, molecular targeted therapy and immunotherapy are also commonly used for a variety of cancers. In patients with advanced or metastatic tumors, systematic therapies with anti-cancer agents are usually fundamental treatment strategies; however, the anti-cancer effects, including prolongation of survival, of such systematic therapies are not always satisfactory in clinical practice. In addition, chemotherapy and molecular targeted therapy results in relatively high frequency of adverse events, especially in elderly patients [1,2,3]. Therefore, many investigators, physicians and patients with cancer are particularly invested in the development of more effective and safer treatment strategies. 

In general, natural products are advantageous because they are easily obtained and relatively safe. In addition, various natural compounds have been reported to be useful to improve the anti-cancer effects of certain chemotherapeutic agents. In recent years, the consideration of natural products as anti-cancer treatments has grown worldwide [4,5,6]. Royal jelly (RJ) is of interest for the improvement of health and medicines. We have also dedicated specific attention to RJ in this review because of following reasons and findings: (1) RJ is special food for queen honeybee in both the larva and adult stages. As a result, it was speculated that RJ prolongs the lifespan of the queen honeybee relative to worker honeybees [7]. (2) RJ can modulate inflammation, oxidative stress and vasodilatory activity [8,9,10,11,12]. These RJ-induced activities are widely considered to be useful to maintain homeostasis and recover from pathological conditions; therefore, RJ has been used as cosmetics, health food, or dietary supplement [13,14], (3) RJ affects the immune system under various physiological and pathological conditions, including malignancies and stimulate not only immunocompetent cells but also antibody production [15,16,17,18,19,20,21]. Thus, RJ is speculated to modulate the wider immune system. (4) RJ has an abundance of the main nutrients, such as proteins, carbohydrates and lipids and has some stronger and specific biological activities compared to other bee products [17,18,19]. From these facts, there is a hypothesis that RJ may have some benefits for cancer treatments. However, we must note the decision of European Food Safety Authority (EFSA) that a cause and effect relationship cannot be established between the consumption of RJ and the claimed effects [20]. On the other hand, this opinion was published in 2011 and then new findings on beneficial effects of RJ for cancer treatments were showed by in vivo and in vitro studies.

In this review, we have first introduced the anti-cancer effects of RJ in in vivo and in vitro studies. Subsequently, we have summarized the usefulness of RJ in the prevention of anti-cancer treatment-induced toxicities in animal models and patients with cancer. In addition, we have shown the molecular mechanisms of the biological activities of RJ in cancer treatment. Finally, therapeutic strategies and the present use of RJ, its future direction and limitations of RJ-related activities have been discussed based on recent publications. 

## 2. Anti-Cancer Effects of Royal Jelly and Its Components

RJ contains water, sugar, proteins and lipids and approximately 90% of RJ lipids are free fatty acids, containing 8–12 carbons that are usually either hydroxyl or dicarboxylic forms [21]. 10-hydroxy-2-decenoic acid (10-HDA), known as a major component of RJ, plays important roles in various biological activities, including inflammation and oxidative stress [22,23]. Therefore, in this section, we have discussed the reported anti-cancer effects of RJ and 10-HDA in various malignancies. 

### 2.1. Anti-Cancer Effects of Royal Jelly 

#### 2.1.1. In Vitro Studies

First, the anti-cancer effects of RJ in cancer cell lines should be considered. Endogenous hormones are closely associated with carcinogenesis, tumor growth and progression in a variety of cancers, such as breast, ovarian and prostate cancer. It is well established estradiol plays crucial roles in the tumor development of breast cancer [24] and it is reported that RJ inhibits estradiol-induced cell proliferation of MCF-7 breast cancer cells [25]. In addition, these anti-cancer effects of RJ were mediated via the suppression of estradiol-related signaling in cell proliferation but not by binding of estradiol to the estrogen receptor [25]. This study also showed that such cancer cell proliferation was inhibited by RJ in the presence of bisphenol, which has environmental estrogen activity, even though RJ did not affect the proliferation in the absence of bisphenol. Essentially, it is speculated that RJ inhibits the proliferative activity of bisphenol in breast cancer cells. Finally, the authors commented that more detailed information on the active substance and physiological properties are important for the clarification of the anti-cancer effects of RJ and we support this opinion. 

In contrast, there are no reports on the relationship between RJ and androgen-related tumor growth in prostate cancer. Similarly, the anti-cancer effect of RJ against hormone-dependent malignant behavior in cervical cancer is unclear, although bee bread, another bee product, was reported to inhibit the tumor growth of HeLa cervical cancer cells [26]. However, the investigation of the effects of RJ in ovarian cancer has still not been performed. Unexpectedly, significantly inhibition of cancer-related characteristics is not commonly reported in the case of RJ monotherapy. For example, although several studies have shown that RJ tended to suppress the tumor growth of astrocytoma, glioblastoma multiforme, astroglia and colorectal cancer cells, these anti-cancer effects are not always recognized to be significant and RJ monotherapy is not recommended [27,28].

#### 2.1.2. In Animal Experiments

In mouse model of breast cancer, orally administered RJ caused significant inhibition of tumor growth as a prophylactic-therapeutic method; however, such anti-cancer effect was not detected when administered following tumor cell inoculation [29]. Briefly, when mice injected subcutaneously with 4T1 mouse mammary tumor cells were treated with RJ for 14 days prior to the transplantation of tumor cells and subsequently for 28 consecutive days, the tumor volume was significantly lower than that in control mice; however, a corresponding inhibitory effect was not found when the mice were treated with RJ for 28 consecutive days after tumor cell transplantation. Thus, RJ intake may be effective as a prophylactic agent but not as a therapeutic agent; thus, the authors suggested that effective administration of RJ might require ≥14 days administration prior to tumor inoculation. 

With regard to relationship between RJ administration and survival, it is reported that the administration of RJ can prolong survival compared to control animals and this effect occurred dose-dependently in a mouse model of Ehrlich ascites tumor [30]. In short, the highest protection conferred an extension of survival of approximately 38%, 71% and 85% for doses of 0.5, 1.0 and 1.5 g/kg for 33 days, respectively. Furthermore, this study suggested that a decrease in prostaglandin E (PGE)-2 might be associated with such anti-cancer effects in addition to immunity-related host resistance [30]. The PGE-2 system is recognized widely as stimulator of carcinogenesis, cancer cell proliferation and invasion and as an inhibitor of apoptosis in various types of cancers [31,32,33]. Furthermore, PGE2 is an important modulator for the activities of immune cells, including macrophages, dendritic cells and natural killer cells [34,35,36]. Thus, there is a possibility that RJ can improve the prognosis via the regulation of malignant aggressiveness and the immune system through the downregulation of PGE-2. 

### 2.2. Anti-Cancer Effects of 10-Hydroxy-2-Decenoic Acid 

**Figure 1:**
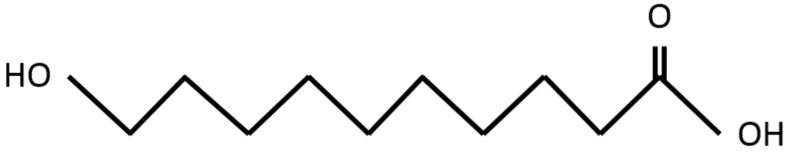
Structure of 10-HAD.

10-HDA (Figure 1) is a major component of RJ and is known to have various biological activities [37]. 

The content of 10-HDA in RJ is reported to be 0.8%–6.5%; moreover, it is known as a unique component of RJ because it is not detected in any other natural raw material, even in other bee products [11,22,38]. To our surprise, anti-cancer effects of 10-HDA in leukemia and ascitic tumors were first reported in approximately 60 years ago [39,40]. In summary, these studies in mice showed that 1.5 mg of 10-HDA per milliliter of cell suspension completely prevented tumor formation in four types of malignant cells: mouse leukemia, 6CSHED lymphosarcoma, the TAS mammary carcinoma and the Ehrlich carcinoma [40]. Unfortunately, this did not prompt further immediate investigation into the anti-cancer effects or biological activities of 10-HDA. However, in recent years, there have been two in vitro studies point out that of the anti-proliferative activity of 10-HDA in colon cancer cells and it is known that the regulation of inflammatory functions or oxidative stress by 10-HDA is an important mediator of these anti-cancer effects [12,28]. In contrast, although we already stated that RJ could inhibit the bisphenol-induced proliferation of breast cancer cells, 10-HDA does not exhibit similar anti-proliferative activity [25]. Thus, the information on the anti-cancer effects of 10-HDA is incomplete and more a detailed analysis at molecular levels in more types of malignancies is necessary to confirm the utility and limitations of 10-HDA as a therapeutic agent. 

## 3. Activity of Royal Jelly against Cancer Therapy-Induced Toxicity

Chemotherapy usually leads to various adverse events, such as bone marrow suppression, gastrointestinal tract disorders, dysfunction of the kidney and the liver, owing to the lack of tumor specificity and the resultant effects on normal tissues. The adverse events caused by systematic cancer therapy are unavoidable, although the symptoms and degrees are dependent on the individual. Decreasing the incidence and severity of adverse events induced by anti-cancer therapies is of great importance for the maintenance of the quality of life (QOL) of patients with cancer. It is also important for the improvement of anti-cancer effects and the prolongation of survival: essentially, some patients are prevented from continuing effect treatment owing to the severe adverse events experienced. It is reported that some anti-cancer agents cause significant adverse events, even when administered at a low dosage [41,42]. Therefore, development of agents that decrease such toxicities is important and currently a major topic of cancer research. In addition, many investigators have given special attention to pharmacotherapy using natural substance as promising future therapeutic options. In this section, the suppressive roles of RJ against cancer therapy-induced toxicities obtained from in vivo studies. 

### 3.1. In Animal Models

Pulmonary fibrosis, one of the most severe adverse events in patients treated with bleomycin (BLM), is associated not only with a reduced QOL but with lethal respiratory discomfort. In rats, cell count and content of pro-inflammatory and pro-fibrotic cytokines in the bronchoalveolar lavage fluid (BALF) were increased by the administration of an intra-tracheal instillation of BLM (7.5 IU/kg); however, such pathological increases were reversed by the oral administration of RJ (50 and 100 mg/kg) for 7 days consecutively prior to BLM administration [43]. In addition to such biochemical markers, they also reported that RJ suppressed histological alterations induced by BLM [43]. Unfortunately, there is little information on the anti-fibrotic effects of RJ or other components in bee honey in BLM-induced pulmonary fibrosis. RJ was also reported to improve serum testosterone level and sperm parameters in rats treated with BLM [44]. The authors speculated that the anti-oxidant properties of RJ exerted positive effects on these parameters [44].

Cisplatin (*cis*-diamminedichloroplatinum; CDDP) is an effective synthetic-spectrum anti-cancer agent and is often included in standard regimens for many solid tumors. However, the clinical usefulness and anti-cancer effects are often restricted owing to the wide variety of adverse effects observed, such as nausea, neurotoxicity, alopecia and fatigue. Nephrotoxicity and hepatotoxicity are the most important of these events [45,46], because they can be fatal to patients with cancer. Consequently, the suppression of these major events caused by CDDP may improve the anti-cancer effect of the drug. With regard to nephrotoxicity, several studies have reported that RJ conferred protective effects on renal function during CDDP treatments in experimental animals [47,48,49]. In short, serum creatinine levels in rats administered a single oral dose of RJ (300 mg/kg) for 15 days consecutively following a single intra-peritoneal injection of CDDP (7 mg/kg) (2.15 ± 0.55 mg/dL) were significantly (*p* < 0.05) lower than those administered CDDP alone (3.15 ± 0.50 mg/dl) [47]. Others report also showed similar results, with pre-treatment (1 h prior to intra-peritoneal administration of 1 mg/kg CDDP kg) with 100 mg/kg RJ reversed the changes in serum parameters, including urea, creatinine and uric acid, observed after CDDP treatment alone [49]. These studies also showed that CDDP led to significant histological changes of congestion, dilatation, epithelial vacuolization and infiltration of some immune cells, mostly macrophages, lymphocytes and plasma cells in the kidney tissues; however, these changes were decreased by RJ [47,49]. In a discussion of the hepatotoxicity, Karadeniz et al. [47] reported that the serum ALT concentration in rats administered CDDP and RJ (29.50 ± 1.70 IU/L) was significantly lower than in rats administered CDDP alone (80.50 ± 2.50 IU/L). The authors commented that such protective functions for the kidney and liver may be due to the anti-apoptotic, anti-oxidant and free radical-scavenging activity of RJ and its compounds [47,48]. Furthermore, RJ suppressed CDDP-induced testicular damage in a rat model [50]. In this study, RJ administration led to a decrease in the malondialdehyde level and an increase in superoxide dismutase, catalase and glutathione-peroxidase activities; in addition, the authors commented that RJ may suppress CDDP-induced sperm toxicity owing to its antioxidant activities.

Cyclophosphamide is a cytotoxic alkylating agent that it is often used for the treatment of cancer. In a rat model, RJ showed significant protective effects against cyclophosphamide-induced prostate cancer damage [51] and oral RJ administration to rats protected against the histological damage to the small intestine induced by methotrexate (MTX), which has anti-cancer effects via folate antagonist activity [52]. In short, mucosal thickness, villus length, villus length/crypt ratio and semi-quantitative histological evaluation in rats treated with MTX was significantly difference to those treated with MTX and RJ [52]. In addition, such protective effects observed in the small intestine after 100 mg/kg RJ administration were higher than those after 50 mg/kg administration [52]. These two different studies showed that part of the protective effects in the prostate and small intestine was potentially associated with the regulation of oxidative stress [51,52].

The compound paclitaxel is extracted from the Pacific yew tree *Taxus brevifolia* and exerts anti-cancer activity via tubulin binding to inhibit the disassembly of microtubules. It is commonly used for conventional therapies and has been the subject of clinical trials for the treatment of various types of malignancies [53]. It is reported that RJ administration protected against paclitaxel-induced histopathological injury, such as diffuse edema, hemorrhage, congestion, hyaline exudates and necrosis and cardiac biomarkers of the creatine kinase level via the suppression of oxidative and nitrosative stress [54].

Unfortunately, there are few reports on the protective effects of RJ against toxicities induced by molecular targeted therapy or immune checkpoint inhibitors in animal models. We believe that more detailed studies about such issues are important.

### 3.2. In Patients with Malignancies

Oral mucositis and gastritis are common adverse events in patients with cancer treated with anti-cancer therapies, including chemotherapy, radiotherapy and molecular targeted therapy [55,56]. It is recognized as one of the most noteworthy adverse events because it may result in a decrease in QOL or the rate of completion of therapy. Various clinical trials on the prevention of mucositis induced by anti-cancer therapies are ongoing [56,57,58]. For example, in patients with head and neck cancer, a randomized single (physician)-blind trial with an RJ-treated group (*n* = 7) and a control group (*n* = 6) was performed to evaluate the clinical utility of RJ for the prevention of oral and esophageal mucositis [59]. In this study, all patients were treated with radiotherapy (66–77 Gy) and chemotherapy (nedaplatin and docetaxel, S-1, or intra-arterial CDDP); in addition, patients in the RJ group took RJ (3 g/day) during radiation therapy. Their results showed that all patients in the control group experienced grade 3 mucositis, which progressed to grade 4 in one patient at 1 month after treatment but that grade 3 mucositis was observed in only 71.4% of patients in the RJ group. In this study, we should note that further studies are needed because of the small sample size and the absence of double blinding. Finally, the authors concluded that prophylactic use of RJ was effective for the reduction of mucositis induced by chemoradiotherapy in these patients.

With regard to the protective effect of CDDP-induced nephrotoxicity, a comparison of the treatments of crude honey, RJ and control was performed in 30 patients with cancer treated with CDDP; randomly divided into the honey group (*n* = 10) and the RJ group (*n* = 10), which were pre-treated before the initiation of CDDP and during CDDP treatment and the control group (*n* = 10) administered CDDP only [60]. This study showed a significant decreased (*p* < 0.05) of serum levels of creatinine and urea before and after treatment in the honey group. However, a similar significant improvement of kidney function parameters was not found in RJ group. In addition, it was shown that a remarkable reduction in these kidney function parameters occurred in 60% of patients in the honey group but only in 40% of patients in the RJ group. The authors speculated that the small sample size may be the explanation for this difference and suggested that further investigation with a larger sample size should be conducted to confirm this issue. In addition, we suggest that a more detailed analysis, including dosage, duration and timing of administration also should be performed. Furthermore, we also speculated that there was the possibility that crude honey, including various honey products such as propolis and bee pollen, is more closely associated with nephroprotective effects more than pure RJ because these products exert protective effects on kidney function [61,62]. Indeed, as mentioned above, animal experiments also showed that the nephroprotective effects of RJ were lower than those of honey [49]. 

A double-blind randomized clinical trial was performed to evaluate the effectiveness RJ on the symptoms of cancer-related fatigue in patients administered anti-cancer therapies [63]. In this trial, 52 patients were invited into two groups: the study group was treated with processed honey and RJ (*n* = 26) and the control group was treated with pure honey (*n* = 26); supplements of 5 mL were administered twice per day for 4 weeks. All three scores of fatigue and performance status in the study group were significantly better than those in control group [63]; however, the authors indicated that further clinical trials with a larger number of patients and a longer duration of intervention are necessary to clarify the role of RJ in managing cancer-related fatigue, because their study populations included a variety of malignancies and treatments. In addition, we should note that honey and RJ but not only RJ, was used in this clinical trial. 

Finally, we have summarized previous reports on the suppression of toxicities by RJ in malignancies in Table 1.

### 3.3. Molecular-Level Changes Induce by Royal Jelly

In this section, we have introduced the molecular mechanism for the prevention of such cancer therapy-induced toxicities (Table 2).

#### 3.3.1. Apoptosis and Proliferation

Many types of chemotherapeutic agents induce apoptosis, not only in malignant cells but also in normal cells. In fact, CDDP remarkably increased the expression of caspase-3, which is a key mediator of apoptosis, in the kidney and liver of rats. However, RJ treatment decreased such caspase-3 reactivity in the proximal tubules of tissues of the kidney and the liver [47]. In contrast, the same study showed that the reactivity of Bcl-xL, which is an inhibitor of apoptosis, was lower in the kidney and liver of rats treated with CDDP than in control rats and that this decrease in reactivity was restored in rats treated with CDDP and RJ [47]. Other investigators have reported that cyclophosphamide increased the expression of Bax, which is a stimulator of apoptosis, in most of the prostatic acini of rats; however, the cyclophosphamide-induced change in Bax expression was improved by the concomitant administration of CDDP and RJ [51]. In addition, this study showed that although CP led to marked morphological changes, such as cystic dilatation with lost papillary fold in acini, flattened lining epithelium, wall integrity and apparent rupture of some acini, these changes of pathological features were suppressed by RJ via the modulation of Bax immunoreactivity [51].

With regard to cell proliferation, there was a report that the expression of bromodeoxyuridine (BrdU), which is commonly used as a marker of cell proliferation, was downregulated in renal tubular epithelial cells by CDDP administration in rats; however, this change was restored by dietary RJ administration [49]. Thus, in animal models, RJ protects from pro-apoptotic activity and the anti-proliferative effects caused by a variety of anti-cancer agents in several normal tissues.

#### 3.3.2. Inflammation and Oxidative Stress 

Inflammation and oxidative stress are closely associated with anti-cancer agent-induced toxicities [67,68]. RJ has important roles in the regulation of inflammation and oxidative stress under various physiological and pathological conditions and the beneficial changes in the relevant molecules are thought to be modulated by RJ [8,9,10,11,12]. 

Oxidative stress is associated with pathological conditions, including disorders of various organs and tissues [69,70]; conversely, anti-oxidants are speculated to prevent oxidative stress-induced damage [71,72]. Glutathione (GSH), glutathione peroxidase (GSH-Px), superoxide dismutase (SOD) and glutathione-S-transferase (GST) are known endogenous anti-oxidants and anti-oxidant parameters [72]; increased levels and activities of these factors were detected in the kidney and the liver of rats treated with CDDP and RJ compared with those administered CDDP alone [47]. In addition, this study also showed that levels of malondialdehyde (MDA), which is commonly used as biomarker of oxidative stress [73], were significantly lower in the kidney or liver tissues of rats treated with CDDP and RJ than those treated with CDDP. Other investigators also showed similar significant changes in GSH and MDA levels in the kidney tissues of mice [66]. Furthermore, it was also reported that RJ decreased the tissue content of MDA, which was increased by an anti-cancer agent, in the lung tissue of rats treated with bleomycin [43]. The GSH-Px level in the prostate was significantly higher in rats administered with cyclophosphamide and RJ than those administered cyclophosphamide only [51] and similar findings was also reported in the lung of rats treated with bleomycin [43]. In rats treated by MTX, the plasma MDA levels in the MTX and RJ-treated group were significantly lower than those in the MTX-treated group and the plasma levels of SOD and GSH-Px in the MTX and RJ-treated group were higher than those in MTX-treated group [52]. Thus, their results support the role of RJ as an anti-oxidant in response to the oxidative stress caused by a variety of anti-cancer agents. 

In the endothelial cells of rat prostate tissues, eNOS expression in the cyclophosphamide and RJ-treated group was significantly lower than that in cyclophosphamide group [51]. Unfortunately, the relationship between such changes and anti-cancer agent-induced toxicities in normal tissues is not well characterized. However, it is possibility that this mechanism may affect the pathological mechanisms of some toxicities owing to the important role of eNOS in endothelial cell survival and angiogenesis [74]. 

With regard to inflammation, it has been reported that cyclophosphamide (CP) increased the serum levels of c-reactive protein (CRP) and tumor necrosis factor (TNF)-α in rats; however, such an increase was not observed in rats orally administered RJ (300 mg) for 14 days by gastric tube, followed by administration of CP [51]. However, a similar result was reported in TNF-α levels in the bronchoalveolar lavage fluid (BALF) of rats treated with bleomycin [43]. Briefly, rats were orally administered RJ orally (50 and 100 mg/kg/day) for 7 days consecutively before the administration of single intratracheal instillation of bleomycin at 7.5 IU/kg and RJ reversed the change in TNF-α levels in BALF. Interestingly, it was also shown that RJ reversed the histopathological alterations induced by BLM and increased the anti-fibrotic cytokine, interferon (IFN)-γ, in BALF that was decreased by BLM [43].

#### 3.3.3. Fibrosis

Fibrosis is closely associated with not only dysfunction of many organs but also tumor progression in various types of cancers [75,76]. Therefore, the appropriate regulation of fibrotic changes by cancer-related factors and anti-cancer therapies is important for the maintenance of homeostasis and to improve the prognosis in patients with cancer. Indeed, many investigators have specifically investigated the molecular mechanisms and preventive strategies of fibrosis in various organs [77,78]. 

However, there are only a few reports of the changes in fibrosis-related factors caused by RJ administration in vivo. One report showed that that intra-peritoneal CDDP administration (1 mg/kg twice weekly for 10 weeks) damaged 60% of renal tubules in rats (Ibrahim) and fibrogenic factors, including α-smooth muscle actin (SMA) in the interstitial tissues and transforming growth factor (TGF)-β1 in renal tubules, were upregulated by this treatment. However, dietary RJ decreased CDDP-induced α-smooth muscle actin and TGF-β1. The induction of such changes at the molecular level by RJ are speculated to confer the protective effects of renal function because such pro-fibrotic changes are closely associated with CDDP-induced nephrotoxicity [77]. Other investigators have also shown that RJ reversed TGF-β levels in the BALF of rats treated with BLM [43]; unexpectedly, they also found that significant activity was detected in in rats orally administered RJ at 50 and 100 mg/kg/day but not at 25 mg/kg/day.

## 4. Therapeutic Strategies for Royal Jelly in the Near Future

Natural products, including RJ, have been investigated for their promise as potential agents for the therapy of patients with malignancies. Indeed, as mentioned above, inhibitory effects, such as the prevention of tumor growth and invasion, have been confirmed by in vivo and in vitro studies. However, many investigators and clinicians feel that the anti-cancer effects exerted by crude RJ alone are far from satisfactory for the required improvements in prognosis and survival. Therefore, various challenges and clinical trials have been performed, as follows. 

Previously, we introduced combined therapies of RJ and other anti-cancer agents. For example, it is reported that the cytotoxic effect of temozolomide (TMZ), which is an alkylating cytostatic drug, in a human glioma cell line was increased by the combination of TMZ and RJ [27]. Briefly, the combination of TMZ with honey, beebread and RJ, exerted stronger cytotoxic activity on human glioblastoma multiforme cells (U87MG) than TMZ alone. In contrast, their results also showed that similar cytotoxic efficacy of the combination therapies was not detected in diffuse astrocytoma cells [27]. Other investigators showed that the combined therapy of RJ and IFN-α demonstrated anti-proliferative activity for the colorectal cancer cell line (CaCo-2) [28]. Interestingly, their study also showed that the highest anti-proliferative activity was obtained when RJ and IFN-α were applied at the ratio 2:1, in a comparison of the ratios of 1:1 or 1:2 [28].

Furthermore, there have been several reports on the anti-cancer and biological effects of mixtures of natural products that contain RJ. For example, GE132+Natural, which is a novel supplement consisting of five compounds (resveratrol, *Ganoderma lucidum*, sulforaphane, lycopene and RJ) showed anti-proliferative effects against cell lines of breast cancer (MCF7), colon cancer (SW480) and prostate cancer (PC-3) in a dose-dependent manner, although it did not affect the proliferation of mesenchymal stem cells and the peripheral blood cell count [79]. Furthermore, there was a report that 100 mg/kg RJ and green tea extracts protected CDDP-induced nephrotoxicity via the restoration of GSH content and MDA production in mice [66]. Thus, a combination of natural products may have suitable anti-cancer effects and protective effects against drug-induced toxicities. However, there is unfortunately little information available from in vivo and in vitro studies or clinical trials. 

Several reports have also suggested that some fractions of RJ are potential therapeutic agents for various types of malignancies. For example, there is a report that among the two protein fractions obtained from the protein extract of RJ precipitated with 30% and 60% ammonium sulfate (called RJP_30_ and RJP_60_, respectively), the survival of human cervical cancer cells (HeLa) was inhibited by RJP_30_ but not by RJP_60_ [80]. More recently, the lipophilic fraction of RJ was reported to have extraordinary anti-proliferative activities in a neuroblastoma cell line (SH-SY5Y) compared with hydrophilic fraction [81]. In addition, this study also found that the biological activities in neuroblastoma cells were stronger than those in immortalized murine myoblasts and prostate cancer cells. Thus, we also agree with their opinion that the search for more effective and disease-specific fractions of RJ may be critical for improvements in the anti-cancer effects. 

At present, various chemical substances and environmental hormones that are present in a variety of plastic products, food and beverage containers and many products in house and work place, are speculated to be associated with tumorigenesis [25,82]. Substances with detoxification activities against such chemicals and hormones have not yet been identified; however, mitigation strategies using natural products are currently under investigation [82]. With regarding RJ, it is reported that RJ has anti-environmental estrogen activity against effects induced by bisphenol A (BPA) in MCF-7 human breast cancer cells. In brief, the number of MCF-7 cells was significantly increased by exposure to 1000 nM BPA for 72 h; however, this increase was inhibited by processed RJ (0.1 g dissolved in 10 mL PBS, centrifuged at 15 kg for 15 min and the top clear layer was used) [82]. 

## 5. Safety of Royal Jelly

RJ is recognized widely as a safe agent in previous studies. Briefly, in a mouse model, the oral administration of 10 g/kg RJ showed no acute toxicity [83]. In addition to acute reactions, RJ administration by gavage did not cause significant changes of serum creatinine, AST and ALT in rats, or alter the histological structure of the kidney and liver [47]. In addition, the same study confirmed that number of apoptotic cells and immunoreactivities of apoptosis-related molecules, such as caspase-3 and Bcl-xL, in the kidney and liver tissues was not changed by RJ. Furthermore, the oral intake of RJ 1.0 g/kg/day for 33 days consecutively did not affect PGE2 production in the supernatant from the peritoneal washes of normal mice [30]. In regard to oxidative stress, RJ was shown to exert no significant influence on MDA and GSH levels in the kidney of mice under physiological conditions [66]. Thus, there is a consensus that RJ is safe as a supplement and drug for clinical use under proper conditions. 

However, we would recommend attention is paid to the following reports. At first, although oral RJ intake did not affect the weight of the lung and the kidney, the weights of the thymus and the spleen were reduced [29]. In this report, the authors commented that changes in the function of the spleen and the thymus by cell-mediated and humoral immunity might be associated with such phenomena and the precise effects of RJ on the immune system required further study [29]. Next, other investigators showed that approximately 10% of the renal tubules had CDDP-induced histopathologic change-like alterations, including moderate changes in rats treated with RJ, although serum parameters of renal function were not significantly altered [49]. Finally, there is the opinion that RJ contains growth factors or hormones that promote the cell growth of adipocytes [80]. These reports do not indicate that RJ has significant toxicities or propensity to cause adverse events; however, more detailed and broader information on the biological effects of RJ at molecular, pathological and clinical levels should be collected for normal cells, tissues and organs.

## 6. Limitations of Studies on Biological Activities of Royal Jelly

The contents of honey are variable and depend on the honeybee subspecies, regional plans and flower pollen [84,85]. Similarly, the biological roles and composition of various fractions of RJ are affected by such factors. Indeed, RJ composition varies by countries; for examples, the percentage of 10-HDA content in Brazil tended to be higher than that in other countries, including Japan, India, Turkey and Switzerland [38]. Furthermore, although it was reported that RJ (0.5–1 mg/mL) enhanced MCF-7 cell proliferation owing to the estrogenic activities of RJ via interaction with estrogen receptor [86], other investigators did not find similar pro-carcinogenic and estrogen-like activities by RJ supplied by other manufacturers [25]. With regard to honey, manuka honey is produced in New Zealand by bees that pollinate the native manuka bush and was shown to prevent CDDP-induced histopathological changes in the liver and suppressed the changes seen in the kidneys; however, Talh honey, one of the most commonly consumed honeys in Saudi Arabia, decreased CDDP-induced liver histopathological changes but had no effect on CDDR-induced kidney changes in model rats [87]. Therefore, it is difficult to compare the protective effects from harmful phenomena and the anti-cancer effects of RJ between different studies. We support the opinion that the influence of these factors should be noted in the discussion and comparison of biological activities of honey products [19]. 

It was also reported that the administration route of RJ affects its anti-cancer effects. For example, in murine mammary carcinoma models, the intraperitoneal or subcutaneous administration of RJ did not affect metastasis formation, whereas intravenous administration prior to tumor cell inoculation significantly inhibited the formation of metastases [88]. In general, RJ is administered orally when used as a supplement and a prophylactic because of simplicity. However, when the clinical trials of RJ-based therapy are planned, the administration method may be an important determinant of its success. In contrast, there is no general agreement on the best administration method to produce the anti-cancer and/or protective effects of RJ in patients with malignancies. Thus, many issues remain to determine the clinical usefulness of RJ in these patients.

## 7. Conclusions

In this review, we have summarized studies on the anti-cancer effects of RJ reported in in vivo and in vitro studies. RJ and its main component, 10-HDA, can inhibit tumor growth and cancer cell invasion via the regulation of various cancer-related factors. In addition, animal experiments have shown that RJ administration leads to prolonged survival with a variety of malignancies. Many reports demonstrated that RJ is useful for protection against anti-cancer agent-induced toxicities, such as mucositis, fibrosis and disorder of the kidney and the liver. Furthermore, the modulation of various biological activities by RJ, including cell survival, inflammation and oxidative stress, is closely associated with the RJ-induced effects. Several clinical studies have confirmed the efficacy of RJ against drug-induced toxicities and clarified the mechanisms in patients with cancer; however, almost all of these clinical trials used relatively small study populations. Therefore, more detailed investigations are essential for a discussion of the clinical utility of RJ in these patients. The efficacy and safety of various combination therapies based on RJ and anti-cancer drugs, using various fractions of RJ, have been reported in vivo and in vitro. Although it is certain many problems remain to be solved, we believe that RJ is a potential tool for the improvement in the QOL and prognosis of patients treated with anti-cancer therapies. 

## Figures and Tables

**Table 1:** Effects of royal jelly on anti-cancer agent-induced toxicities.

Toxicities	Agents	Objective	Summary of Effects	Reference
**Pulmonary Fibrosis**	Bleomycin	Rat	Attenuated oxidative damage and fibrosis	[43]
**Oral** **Mucositis**	5-Fluorouracil	Hamsters	Ointments significantly and dose-dependently improved the recovery from damage	[64]
	Radiation and chemotherapy	One hundred and three patients	Improved the signs of oral mucositis and shortened its healing time	[65]
	Chemoradiation therapy	Thirteen head and neck cancer patients	Reduced the toxicity in by RJ in randomized clinical trials.	[59]
**Intestinal Damage**	Methotrexate	Rats	Suppressed damage through an increase in the activities of anti-oxidant factors	[52]
**Cardio-** **Toxicity**	Paclitaxel	Rats	Conferred protection against histopathological and biochemical alterations	[54]
**Nephro-Toxicity**	Cisplatin	Rats	Inhibited elevation of serum creatinine and prevented histological alterations.	[47]
	Cisplatin	Rats	Histopathologic findings and oxidative parameters were partially reversed	[48]
	Cisplatin	Rats	Reversed the changes in serum creatinine, urea and uric acid.	[49]
	Cisplatin	Thirty-two patients	Serum creatinine and urea were not changed before and after the treatment.	[60]
**Hepato-Toxicity**	Cisplatin	Rats	Inhibited elevations of serum markers and histological alterations.	[47]
**Fatigue**	Hormone therapy, chemotherapy and radiotherapy	Fifty-two patients	Ameliorated toxicity in a double-blind randomized study	[63]
**Testis Damage/Fertility**	Cisplatin	Rats	Histopathologic findings in the testes were partially reversed	[48]
	Bleomycin	Rats	Improved serum levels of testosterone and sperm parameters	[44]
**Prostate Damage**	Cyclophosphamide	Rats	Protected against drug-induced prostate tissue damage.	[51]

**Table 2:** Molecular-level changes induced by royal jelly in response to anti-cancer therapies.

Molecules	Organs	Species	Agents	Change	Reference
Apoptosis					
Bax	Prostate	Rats	Cyclophosphamide	↓	[51]
Bcl-xL	Kidney, liver	Rats	Cisplatin	↑	[47]
Caspase-3	Kidney, liver	Rats	Cisplatin	↓	[47]
Proliferation					
BrdU	Kidney	Rats	Cisplatin	↑	[49]
Inflammation					
CRP	Serum	Rats	Cyclophosphamide	↓	[51]
TNF-α	Serum	Rats	Cyclophosphamide	↓	[51]
	BALF	Rats	Bleomycin	↓	[43]
Oxidative stress					
eNOS	Prostate	Rats	Cyclophosphamide	↓	[51]
GSH	Kidney	Mice	Cisplatin	↑	[66]
	Kidney, liver	Rats	Cisplatin	↑	[47]
GSH-Px	Prostate	Rats	Cyclophosphamide	↑	[51]
	Kidney, liver	Rats	Cisplatin	↑	[47]
	Lung	Rats	Bleomycin	↑	[43]
	Plasma	Rats	Methotrexate	↑	[52]
GST	Kidney, liver	Rats	Cisplatin	↑	[47]
MDA	Kidney	Mice	Cisplatin	↓	[66]
	Kidney, liver	Rats	Cisplatin	↓	[47]
	Lung	Rats	Bleomycin	↓	[43]
	Plasma	Rats	Methotrexate	↓	[52]
SOD	Kidney, liver	Rats	Cisplatin	↑	[47]
	Plasma	Rats	Methotrexate	↑	[52]
Fibrosis					
α-SMA	Kidney	Rats	Cisplatin	↓	[49]
IFN-γ	BAL	Rats	Bleomycin	↑	[43]
TGF-β1	Kidney	Rats	Cisplatin	↓	[49]
	BALF	Rats	Bleomycin	↓	[43]

Bax, Bcl2-associated X protein; Bcl-xL, B-cell lymphoma-extra-large; BrdU, Bromodeoxyuridine; CRP, C-reactive protein; TNF, tumor necrosis factor; BALF, bronchoalveolar lavage; eNOS, nitric oxide synthase; GSH, glutathione; GSH-Px, glutathione peroxidase; GST, glutathione-S-transferase; MDA, malondialdehyde; SOD, superoxide dismutase; SMA, smooth muscle actin; IFN, interferon; TGF, transforming growth factor. ↓, Decreased molecular change by chemotherapeutic agents; ↑, Increased molecular change by chemotherapeutic agents.
